# Trends in Health-Related Quality of Life Among Adolescents in the United States, 2001–2010

**DOI:** 10.5888/pcd10.120334

**Published:** 2013-07-03

**Authors:** Wanjun Cui, Matthew M. Zack

**Affiliations:** Author Affiliation: Matthew M. Zack, Centers for Disease Control and Prevention, Atlanta, Georgia.

## Abstract

Health-related quality of life (HRQOL) measures are often used to track changes in population health, mostly among adults. Prompted by the recent US recession, we assessed trends in adolescent HRQOL by using cross-sectional data from the 2001–2010 National Health and Nutrition Examination Survey. Adolescents’ self-rated health and reported mental health declined significantly, especially among those in low-income families, but their physical health and activity limitation did not change. Because these HRQOL declines occurred at the end of the decade and especially among adolescents from low-income families, we conclude that these declines are consistent with recession effects and warrant further study.

## Objective

Health-related quality of life (HRQOL) is an individual’s or group’s perceived physical or mental health over time (1). The Centers for Disease Control and Prevention (CDC) has developed a set of standard HRQOL measures to monitor population health and develop policies to promote public health (2–4). Adults’ HRQOL varies with demographic and socioeconomic characteristics, chronic diseases, risky and protective behaviors, and time (1,2). However, much less is known about adolescents’ HRQOL. This study aimed to assess trends in HRQOL in a population-based sample of US adolescents. It also examined the effect of family income level on adolescent HRQOL.

## Methods

The 2001–2010 National Health and Nutrition Examination Survey (NHANES), a nationally representative cross-sectional survey to ascertain the health and nutritional status of the noninstitutionalized US civilian population, provided data for this study (5). At the NHANES mobile examination center, 7,087 adolescents aged 12 to 17 were interviewed, and approximately 93% of them answered questions about their HRQOL.

The HRQOL measures include self-rated health and number of physically unhealthy days, mentally unhealthy days, and activity limitation days (2). The self-rated health measure asks the question “Would you say that in general your health is excellent, very good, good, fair, or poor?” We grouped responses into fair or poor health, good health, and very good or excellent health. The measures for unhealthy days asked respondents how many days during the past 30 days their physical or mental health was not good or their physical or mental health limited them from doing usual activities. We grouped responses for these separate measures into 0 days, 1 to 13 days, and 14 to 30 days.

The main independent variable was the interview year of the 5 NHANES survey cycles (2001–2002, 2003–2004, 2005–2006, 2007–2008, and 2009–2010). To assess whether adolescents’ HRQOL varied by family income, we created an interaction term between interview year and family poverty–income ratio. We defined low income as 130% or less of federal poverty guidelines, middle income as more than 130% to 350%, and high income as greater than 350%. We adjusted for possible confounding from sex, race/ethnicity, age, physical inactivity, and cigarette smoking.

We used multinomial logistic regression in SAS-callable SUDAAN 10.0 (6), accounting for NHANES’ complex sample survey design, to estimate both unadjusted percentages (not reported) and percentages adjusted for potential confounders and their 95% confidence intervals (CIs) for the HRQOL outcomes by interview year alone and the interaction between interview year and family poverty–income ratio. We used *t* tests to detect significant differences between percentages and logistic regression to test for trends.

## Results

Overall, adolescents’ self-rated health was fairly stable from 2001 through 2004 but worsened afterward. The adjusted percentage of adolescents reporting excellent or very good health in 2009–2010 (51.8%) was significantly lower than in 2001–2002 (63.4%) and 2003–2004 (64.0%) (Table 1). The adjusted percentage of adolescents reporting fair or poor health was significantly higher in 2007–2008 (10.0%) than in 2003–2004 (5.7%). This same pattern occurred among adolescents with low family income (Table 2). The percentage of adolescents from high-income families who reported excellent or very good health also decreased, but the percentage who reported fair or poor health did not increase.

**Table 1:** Adjusted^a^ Percentages of the CDC’s HRQOL Measures Among Adolescents, by Interview Year, National Health and Nutrition Examination Survey 2001–2010

Year	N^b^	% (95% CI)	% (95% CI)	% (95% CI)
**Self-Rated Health**				
**Year**	**N**	**Excellent/Very Good**	**Good**	**Fair/Poor**
All years	5,294	58.0 (56.0–60.0)	33.7 (31.9–35.5)	8.3 (7.1–9.5)
2001–2002	1,323	63.4 (58.7–68.1)	30.2 (26.6–33.9)	6.3 (4.4–8.3)
2003–2004	1,234	64.0 (59.9–68.2)	30.3 (26.1–34.4)	5.7 (4.4–7.0)
2005–2006	1,276	55.3 (51.9–58.7)	35.9 (32.8–39.1)	8.7 (6.3–11.2)
2007–2008	687	56.3 (51.2–61.4)	33.7 (29.8–37.7)	10.0 (7.2–12.8)
2009–2010	774	51.8 (47.5–56.2)	37.9 (33.2–42.6)	10.3 (6.7–13.9)
**Physically Unhealthy Days**				
**Year**	**N**	**0 Days**	**1–13 Days**	**14–30 Days**
All years	5,291	61.3 (59.4–63.3)	34.7 (32.7–36.6)	4.0 (3.2–4.8)
2001–2002	1,322	61.2 (57.9–64.6)	34.5 (31.2–37.7)	4.3 (3.2–5.4)
2003–2004	1,234	66.1 (62.3–70.0)	29.6 (25.4–33.7)	4.3 (2.4–6.1)
2005–2006	1,275	64.4 (61.2–67.5)	32.2 (29.0–35.4)	3.5 (1.9–5.0)
2007–2008	686	57.6 (53.0–62.2)	37.7 (32.8–42.7)	4.7 (2.4–6.9)
2009–2010	774	57.5 (52.1–63.0)	39.1 (33.7–44.5)	3.4 (1.5–5.2)
**Mentally Unhealthy Days**				
**Year**	**N**	**0 Days**	**1–13 Days**	**14–30 Days**
All years	5,288	57.4 (55.4–59.4)	35.7 (33.8–37.6)	6.9 (5.9–7.9)
2001–2002	1,321	60.6 (56.1–65.1)	33.9 (29.0–38.9)	5.5 (4.1–6.8)
2003–2004	1,234	64.0 (59.1–68.8)	30.9 (27.3–34.6)	5.1 (3.1–7.1)
2005–2006	1,274	60.9 (57.8–64.1)	33.3 (30.5–36.0)	5.8 (3.8–7.8)
2007–2008	686	52.6 (47.9–57.3)	38.8 (34.6–43.0)	8.6 (5.4–11.7)
2009–2010	773	49.4 (45.6–53.2)	41.0 (36.3–45.7)	9.6 (7.2–12.0)
**Activity Limitation Days**				
**Year**	**N**	**0 Days**	**1–13 Days**	**14–30 Days**
All years	5,290	79.0 (77.2–80.9)	18.7 (17.0–20.4)	2.3 (1.6–2.9)
2001–2002	1,321	79.5 (76.5–82.5)	19.3 (16.8–21.8)	1.2 (0.3–2.1)
2003–2004	1,234	83.6 (80.0–87.2)	15.4 (11.8–19.0)	1.0 (0.3–1.8)
2005–2006	1,276	81.1 (78.7–83.5)	17.1 (14.8–19.5)	1.8 (1.0–2.7)
2007–2008	686	77.3 (71.9–82.6)	17.4 (13.2–21.5)	5.4 (2.7–8.0)
2009–2010	773	73.9 (68.7–79.0)	24.3 (19.2–29.3)	1.9 (0.8–2.9)

Abbreviations: CDC, Centers for Disease Control and Prevention; HRQOL, health-related quality of life; CI, confidence interval.

a Model adjusted for demographic characteristics (sex, race/ethnicity, age, and family poverty-income ratio) and risky behaviors (physical inactivity and cigarette smoking).

b Sample sizes exclude missing values.

**Table 2:** Adjusted^a^ Percentages of the CDC’s HRQOL Measures Among Adolescents, by Interview Year for Level of Family Income, National Health and Nutrition Examination Survey 2001–2010

Year	Low-Income Family, % (95% CI)			Middle-Income Family, % (95% CI)			High-Income Family, % (95% CI)		
**Self-Rated Health**									
**Year**	**Excellent/Very Good**	**Good**	**Fair/Poor**	**Excellent/Very Good**	**Good**	**Fair/Poor**	**Excellent/Very Good**	**Good**	**Fair/Poor**
2001–2002	55 (47–63)	37 (30–45)	8 (5–12)	66 (59–73)	29 (23–34)	5 (3–10)	67 (61–73)	27 (21–33)	6 (3–11)
2003–2004	60 (52–67)	34 (27–42)	6 (3–9)	60 (53–66)	34 (27–41)	7 (4–10)	72 (63–80)	24 (16–33)	5 (2–10)
2005–2006	52 (48–56)	38 (33–44)	10 (6–14)	50 (41–58)	37 (31–44)	13 (9–18)	64 (59–69)	33 (28–37)	3 (1–7)
2007–2008	51 (45–58)	36 (29–44)	12 (8–18)	53 (44–61)	35 (29–41)	13 (8–20)	64 (55–72)	32 (23–42)	5 (1–11)
2009–2010	46 (38–53)	42 (34–50)	12 (8–19)	54 (48–60)	34 (26–43)	12 (7–19)	54 (47–61)	39 (33–46)	7 (3–12)
**Physically Unhealthy Days**									
**Year**	**0 Days**	**1–13 Days**	**14–30 Days**	**0 Days**	**1–13 Days**	**14–30 Days**	**0 Days**	**1–13 Days**	**14–30 Days**
2001–2002	63 (57–69)	32 (26–38)	5 (2–9)	61 (57–66)	35 (29–41)	4 (2–7)	60 (51–68)	36 (29–43)	5 (2–8)
2003–2004	70 (63–76)	26 (20–33)	4 (2–8)	68 (62–74)	27 (22–33)	5 (2–11)	62 (53–70)	34 (25–44)	4 (2–8)
2005–2006	65 (58–72)	30 (22–39)	5 (2–11)	63 (57–68)	34 (30–39)	3 (1–6)	65 (61–70)	32 (28–36)	3 (1–7)
2007–2008	54 (49–59)	40 (35–46)	5 (3–9)	64 (55–72)	31 (24–40)	5 (2–10)	54 (44–64)	41 (32–52)	4 (1–10)
2009–2010	61 (53–68)	35 (30–41)	4 (1–9)	59 (52–66)	38 (32–45)	3 (1–8)	53 (42–65)	43 (32–55)	3 (1–8)
**Mentally Unhealthy Days**									
**Year**	**0 Days**	**1–13 Days**	**14–30 Days**	**0 Days**	**1–13 Days**	**14–30 Days**	**0 Days**	**1–13 Days**	**14–30 Days**
2001–2002	59 (53–65)	35 (30–41)	5 (2–10)	61 (52–69)	35 (27–44)	4 (2–6)	61 (56–66)	32 (25–39)	7 (4–12)
2003–2004	63 (55–70)	30 (22–39)	7 (3–12)	66 (58–73)	28 (23–34)	6 (2–13)	63 (57–69)	34 (28–40)	3 (1–8)
2005–2006	55 (49–61)	37 (31–43)	8 (5–12)	60 (54–65)	34 (29–39)	6 (3–12)	66 (60–72)	30 (25–36)	3 (1–7)
2007–2008	52 (43–60)	43 (35–52)	5 (3–8)	56 (47–65)	32 (25–40)	11 (7–18)	50 (41–60)	41 (34–48)	9 (4–16)
2009–2010	46 (41–51)	43 (37–49)	11 (7–17)	45 (38–53)	45 (38–51)	10 (6–16)	56 (47–65)	36 (26–48)	8 (4–13)
**Activity Limitation Days**									
**Year**	**0 Days**	**1–13 Days**	**14–30 Days**	**0 Days**	**1–13 Days**	**14–30 Days**	**0 Days**	**1–13 Days**	**14–30 Days**
2001–2002	81 (73–87)	17 (12–24)	2 (0–8)	82 (77–86)	18 (14–22)	1 (0–3)	76 (72–80)	23 (19–27)	1 (0–4)
2003–2004	84 (79–88)	15 (10–20)	1 (0–4)	83 (75–89)	16 (10–24)	1 (0–4)	84 (77–89)	15 (10–22)	1 (0–4)
2005–2006	85 (79–90)	13 (8–20)	2 (0–5)	79 (73–84)	18 (13–24)	3 (1–6)	81 (74–86)	19 (14–25)	1 (0–3)
2007–2008	75 (66–82)	19 (14–26)	6 (2–11)	79 (68–87)	16 (10–24)	5 (1–13)	78 (69–85)	17 (11–26)	5 (2–12)
2009–2010	74 (68–79)	24 (19–29)	2 (1–5)	71 (62–79)	27 (20–36)	2 (0–5)	77 (68–84)	21 (14–30)	2 (0–4)

Abbreviations: CDC, Centers for Disease Control and Prevention; HRQOL, health-related quality of life; CI, confidence interval.

a Model adjusted for demographic characteristics (sex, race/ethnicity, and age) and risky behaviors (physical inactivity and cigarette smoking).

The adjusted percentage of adolescents reporting zero, 1 to 13, or 14 or more physically unhealthy days did not change over the study period (Table 1) or by family income level (Table 2).

Adolescents’ mental health worsened over time, especially recently. The adjusted percentage reporting zero mentally unhealthy days was fairly stable from 2001 through 2006 but declined significantly from 60.9% in 2005–2006 to 49.4% in 2009–2010 (Table 1). Yet, only in adolescents from low-income families did the percentage of zero mentally unhealthy days significantly decrease (from 63% in 2003–2004 to 46% in 2009–2010). The percentage reporting 14 to 30 mentally unhealthy days increased significantly, almost doubling, from 2001–2004 through 2009–2010. However, the adjusted percentage of adolescents who reported 14 to 30 mentally unhealthy days increased significantly only among adolescents from low-income families (from 5% in 2007–2008 to 11% in 2009–2010) and middle-income families (from 4% in 2001–2002 to 10% in 2009–2010); it did not increase significantly among adolescents from high-income families (Table 2). The adjusted percentage of zero activity limitation days was significantly higher in 2003–2004 than in 2009–2010, but the percentage of 1 or more activity limitation days did not increase (Table 1). Among adolescents from low-income families the percentage of zero activity limitation days also significantly decreased between 2005–2006 and 2009–2010 (Table 2).

## Discussion

This study is the first to assess recent trends in HRQOL using CDC’s HRQOL measures among population-based samples of adolescents. Adolescents’ self-rated health and mental health generally worsened over the study period, especially more recently, but the number of physically unhealthy days and recent activity limitation days did not change consistently. Because the worsening occurred more recently, the 2008–2009 US recession might have adversely affected adolescent HRQOL. Although NHANES data do not provide direct information on recession effects (for example, parental unemployment), we found greater worsening of HRQOL among adolescents from low- and middle- income families than among those from high-income families. The recession more plausibly explains this recent worsening of HRQOL and concerns about future economic opportunities than other explanations such as the wars in Afghanistan or in Iraq that began several years earlier. Although poor adolescent HRQOL is associated with individual risky behaviors such as cigarette smoking and physical inactivity (7–9), adjusting for these potential confounders did not affect the observed HRQOL trends.

Our study has several limitations. First, because our analyses covered only 5 biennial cycles, we could not detect whether adolescent HRQOL improved as the economic situation improved more recently. Second, NHANES data are self-reported and subject to misclassification, although the CDC’s HRQOL measures appear valid and reliable among adolescents (10). Third, because NHANES is cross-sectional and not longitudinal, compositional changes to the sample across survey cycles might have reduced our ability to detect trends in this study.

Because adolescence can be challenging and stressful, and because risky behaviors may accompany poor adolescent HRQOL (11), this study’s findings suggest a need to develop policies that help adolescents, especially those from low-income families, to maintain if not improve their HRQOL.
